# A viscous quantum hydrodynamics model based on dynamic density functional theory

**DOI:** 10.1038/s41598-017-14414-9

**Published:** 2017-11-10

**Authors:** Abdourahmane Diaw, Michael S. Murillo

**Affiliations:** Department of Computational Mathematics, Science and Engineering, Michigan State University East Lansing, Michigan, 48823 USA

## Abstract

Dynamic density functional theory (DDFT) is emerging as a useful theoretical technique for modeling the dynamics of correlated systems. We extend DDFT to quantum systems for application to dense plasmas through a quantum hydrodynamics (QHD) approach. The DDFT-based QHD approach includes correlations in the the equation of state self-consistently, satisfies sum rules and includes irreversibility arising from collisions. While QHD can be used generally to model non-equilibrium, heterogeneous plasmas, we employ the DDFT-QHD framework to generate a model for the electronic dynamic structure factor, which offers an avenue for measuring hydrodynamic properties, such as transport coefficients via x-ray Thomson scattering.

## Introduction

Access to high-power laser sources, such as the Linac Coherent Light Source (LCLS)^1^, National Ignition Facility (NIF)^2^ and Omega Laser^3^, has opened the path to investigating essential properties of non-ideal plasmas such as ionization potential depression^4^, transport coefficients^5^ and ionization state^6^. Understanding the dynamical properties of non-ideal plasmas is critical for modeling and designing high energy-density science experiments, including inertial-confinement fusion^7^, cluster explosions^8^, laser-produced ion beams^9^, hypervelocity impacts^10^, in nanotechnology^11,12^ and astrophysics^13^.

Among all the approaches to modeling heterogeneous, non-equilibrium quantum systems, quantum hydrodynamics (QHD) is a computationally attractive approach with rich history in statistical mechanics. Shortly after the development of quantum mechanics, Bloch^14^ proposed the first QHD model by simply choosing the Thomas-Fermi pressure for the electrons in an otherwise classical hydrodynamics model. In 1964, Hohenberg and Kohn^15^ developed ground-state density functional theory for the inhomogeneous electron gas, which was immediately generalized to finite temperature by Mermin^16^. Combining the ideas of Bloch with DFT, Ying^17^ proposed a new quantum hydrodynamic model via an adiabatic generalization of the density functionals. In Ying’s model, the pressure is represented by *P*[*n*(*r,t*)] with *n*(*r,t*) a time-dependent density described by the continuity equation. Ying’s QHD model includes explicitly all correlation and exchange effects included in the chosen energy functional. Using an alternate approach, Gasser and Jüngel^18^ derived QHD equations using the Schrodinger equation with Wentzel-Kramers-Brillouin (WKB) wave functions. This approach yields the classical momentum equation with the Bohm potential but it does not account for correlations. Correlations effects and quantum degeneracy can be included in an *ad hoc* manner in this model by replacing the Bohmian potential with quantum potentials^12^ or self-consistently through orbital-free density functional theory (OF-DFT)^19,20^. In yet another approach, using the moment expansion of the Wigner-Boltzmann equation, Gardner^21^ proposed a QHD model for semiconductor devices that extends the classical hydrodynamic model to include $O({\hslash }^{2})$ quantum corrections. Similar results were obtained with the Wigner-Poisson system by Manfredi and Haas^22^ for a quantum electron gas. Following Levermore^23^, Degond and Ringhofer^24^ used a non-commutative version of the entropy externalization principle to build a QHD model starting from the quantum Liouville equation. The moment equations are closed by a quantum Wigner distribution function that minimizes the entropy.

Despite these important advances, describing collisional processes in moderately coupled quantum plasmas remains a challenge^10,22,25^. Here, we explore an alternate approach based on a new formulation of quantum hydrodynamics (QHD). QHD approaches have the advantage of including equation-of-state and transport quantities more naturally than response-function approaches. Apart from these potential modeling advantages, QHD models of DSF therefore also provide access to experimental measurements of these quantities, thereby extending the utility of DSF. We develop a QHD framework based on the extension of the classical dynamical density functional theory (DDFT)^26–28^, a variant of time-dependent density functional theory (TDDFT)^29^. DDFT provides a set of hydrodynamics equations by taking the velocity moments of Liouville equation and closes the system using density functional theory^26–28^. A fundamental assumption of this theory is that the equilibrium energy functional of the system can be used to guess the correlation energy functional when the system is out of equilibrium. While DDFT has found wide use in many-body classical systems^30–32^, we extend its use in quantum systems^17^ to viscous quantum systems, in general, and to DSF, specifically.

We apply the DDFT-QHD model to stationary, homogeneous and isothermal plasmas for which the dynamic structure factor (DSF) is well defined. While the DSF is of interest in its own right, it is also connected to x-ray Thomson scattering (XRTS) experiments; XRTS yields much essential information about plasmas, including density, temperature and atomic physics information (e.g., ionization state^6^, ionization potential depression^4^, etc.).

## Results

Dense strongly coupled plasmas are characterized by large collisional contributions and degenerate electrons. These features make the DDFT-QHD approach a reliable tool for accurately describing the dynamical properties of these systems. For simplicity, here, we consider a quantum plasma comprising only electrons with density distribution *n* and mass *m* interacting through a pairwise Coulomb potential $v(|{\bf{r}}-{\bf{r}}\text{'}|)$. We use atomic units (i.e., $e=m=\hslash =4\pi {\varepsilon }_{0}=1$) for the remainder of this work. The hydrodynamic equations for the electrons can be written generally as

1 $$\frac{\partial n}{\partial t}+\nabla \cdot (n{\bf{u}})=\mathrm{0,}$$

2 $$\frac{\partial (n{\bf{u}})}{\partial t}+\nabla \cdot (n{\bf{uu}})=-\nabla \cdot  {\mathcal F} ,$$

which are continuity and momentum equations written in terms of a generalized force tensor ${\mathcal F}$. Note that the continuity equation (1) and the left-hand side of (2) are generic, with the physical properties of the quantum electron gas entering through terms on the right-hand side of (2). In the DDFT approach^17,28,33^, it is assumed that the system is close enough to equilibrium that an adiabatic closure can be chosen for ${\mathcal F}$; that is, ${\mathcal F} = {\mathcal F} [n,{\bf{u}}]$. The primary assumption of this model is that the system is near equilibrium, a condition well satisfied in highly collisional plasmas. Further, the equilibrium density is forced to be consistent with the thermodynamic ground state of the system by choosing the diagonal portion of the tensor to be of the form $\delta {\rm{\Omega }}[n]/\delta n$, where Ω is the free energy of the system. When ${\rm{\Omega }}[n]$ is expressed using orbital-free density-functional theory (OFDFT), that portion of ${\mathcal F}$ is closed. The off-diagonal portion of ${\mathcal F}$ can be written in its long-wavelength form to yield a generalized Navier-Stokes equation of the form

3 $$-\nabla \cdot  {\mathcal F} ,=-n\nabla \frac{\delta {\rm{\Omega }}[n]}{\delta n}+\eta \nabla \cdot \nabla {\bf{u}}+(\xi +\frac{\eta }{3})\nabla (\nabla \cdot {\bf{u}}),$$

where *η* is the shear viscosity, and *ξ* is the bulk viscosity; all other symbols have their usual meanings. Provided *η* and *ξ* can be expressed in terms of $(n,{\bf{u}})$, the hydrodynamic equations are closed.

In DDFT, one writes the total free-energy functional as

4 $${\rm{\Omega }}[n]=T[n({\bf{r}},t)]+{{\rm{\Omega }}}_{H}[n]({\bf{r}},t)+{{\rm{\Omega }}}_{xc}[n({\bf{r}},t)],$$

where $T[n({\bf{r}},t)]$ is the free energy of the noninteracting system, ${\Omega }_{H}[n]({\bf{r}},t)$ is the Hartree free-energy functional, and ${{\rm{\Omega }}}_{xc}[n]({\bf{r}},t)$ is the exchange-correlation (xc) functional. The Hartree term is exactly known and is an explicit function of space and time.

A key advantage to the DDFT approach to QHD is that all thermodynamic properties are included self-consistently through the total free energy Ω, for which a wide range of approximations are available^34–37^. In fact, this approach is very similar to the well-known generalized hydrodynamics, developed by Frenkel^38^, that extends the classical Navier-Stokes equation to describe the properties of both solid and liquid bodies. Furthermore, our DDFT-QHD approach can be connected with other approaches based on Bohmian dynamics. If we set the viscous terms equal to zero in (3) and choose the gradient-corrected Thomas-Fermi (TF) functional for *T*[*n*], one recovers the well-known Bohmian QHD^20^ form; again, however, the DDFT approach enforces self-consistency of its form with the other terms in the free energy. The connection between DFT and the Bohmian potential will be briefly shown below.

Density fluctuations are not readily available in density-functional theories, and our DDFT-QHD approach suffers from this limitation. However, in equilibrium, the fluctuation-dissipation theorem allows us to connect the linear response of the system to density fluctuations. We write the DSF of the electrons as

5 $${S}_{{\rm{e}}e}({\bf{k}},\omega )=-\frac{1}{\pi }\frac{{\rm{I}}m{\chi }_{{\rm{e}}e}({\bf{k}},\omega )}{1-{e}^{-\beta \omega }},$$

where *β* is the inverse electron temperature and ${\chi }_{{\rm{e}}e}({\bf{k}},\omega )$ is the susceptibility of the free electrons. A large body of literature^39^ focuses on the calculation of the system DSF $S(k,\omega )$, with most work based on the Chihara^40^ decomposition

6 $$S(k,\omega )=|f(k)+q(k{)|}^{2}{S}_{ii}(k,\omega )+{Z}_{b}\int d\omega ^{\prime} {S}_{s}(k,\omega ^{\prime} ){S}_{ce}(k,\omega -\omega ^{\prime} )+{Z}_{f}{S}_{{\rm{e}}e}(k,\omega \mathrm{).}$$

The quantities $f(k)$ and $q(k)$ are the Fourier components of the density of bound and free electrons. The first term of (6) corresponds to low-frequency electron-density fluctuations arising from ion dynamics and is proportional to the ion-ion DSF ${S}_{ii}(k,\omega )$. The factor ${S}_{ce}(k,\omega )$ in the second term describes the contribution from core electrons^41^ and is modulated by the ion self-motion ${S}_{s}(k,\omega ^{\prime} )$. The third term is the free-electron DSF ${S}_{{\rm{e}}e}(k,\omega )$ in the presence of a uniform ionic background. The quantity ${S}_{{\rm{e}}e}(k,\omega )$ can be obtained from the standard Lindhard dielectric function within the random-phase approximation (RPA), or extended to include collisions as proposed by Mermin^42^. Thiele *et al*. generalized the Mermin form to include a dynamic collision frequency within the Born approximation^43^, and Arkhipov *et al*. generalized the Mermin form to two-component plasmas, including sum rules^44^.

The RPA results were also improved by including exchange and correlations through the local field corrections^45–52^. The ionic correlations contributions in a warm dense matter have been considered by Gregori and Gericke^53^. In this scheme, the strongly coupled effects of the ions are included through the different components of the memory function constrained by the sum rules^54^. This phenomenological approach has been applied successfully in Coulomb liquid^54–57^ community for systems where the memory functions have a Gaussian or exponential form. However, for more complex systems, the form of the memory becomes mathematically intractable. Schmidt and coworkers^58^ have proposed a hydrodynamic model that begins with moments of the Wigner-Poisson system with a collision term added. In such an approach you cannot describe correlations properly since the resulting pressure term is of an *ideal gas*. The DDFT-QHD approach we introduce here accounts for self-consistently many-body physics effects and also non-local hydrodynamic effects through the choice of the free-energy functional.

The linear susceptibility associated with a weak external potential $\delta {v}_{ext}({\bf{k}},\omega )$ that induces a disturbance $\delta n({\bf{k}},\omega )$ in the electronic density $n({\bf{k}},\omega )$ is defined as

7 $${\chi }_{{\rm{e}}e}({\bf{k}},\omega )=\frac{\delta n({\bf{k}},\omega )}{\delta {v}_{ext}({\bf{k}},\omega )}\mathrm{.}$$

Thus, the susceptibility can be determined by linearizing the quantum hydrodynamics equation and using (7). To do, we first expand the density and velocities about a uniform mean as

8 $$n({\bf{r}},t)={n}_{0}+\delta n({\bf{r}},t),$$

9 $${\bf{u}}({\bf{r}},t)=\delta {\bf{u}}({\bf{r}},t),$$

which yields the linearized QHD equations in Fourier space:

10 $$-\omega \delta n+{n}_{0}{\bf{k}}\cdot \delta {\bf{u}}=\mathrm{0,}$$

11 $$-{n}_{0}\omega \delta {\bf{u}}=-{\bf{k}}{n}_{0}\frac{\delta \tilde{V}}{\delta n}{|}_{0}\delta n+i{k}^{2}(\xi +\frac{4}{3}\eta )\delta {\bf{u}}+{\bf{k}}{n}_{0}\delta {v}_{{\rm{e}}xt},$$

where $V({\bf{r}})=\delta {\rm{\Omega }}[n]/\delta n$, and the tilde sign denotes the Fourier transform. By combining (10) and (11) and using (7), we obtain an expression for the electron susceptibility:

12 $${\chi }_{{\rm{e}}e}(k,\omega )={k}^{2}{n}_{0}-{\omega }^{2}+{k}^{2}{n}_{0}\frac{\delta \tilde{V}({\bf{k}})}{\delta n}{|}_{0}-i\frac{{k}^{2}{\eta }_{l}}{{n}_{0}}\omega ,$$

where ${\eta }_{l}=\mathrm{(4}\eta /3+\xi )$ is the longitudinal viscosity. To proceed, we need to choose specific forms for the different contributions of the free-energy functional Ω[*n*]. The free-energy functional is typically chosen to ensure that an accurate equilibrium density is recovered, although exact analytical forms are generally not known. However, the contributions of the excess free-energy functional to the free energy of the system, ${{\rm{\Omega }}}_{ex}[n]={{\rm{\Omega }}}_{H}[n]+{{\rm{\Omega }}}_{xc}[n]$, can be expressed formally in terms of the direct correlation function ${c}_{{\rm{e}}e}(|{\bf{r}}-{\bf{r}}{\boldsymbol{^{\prime} }}|)$ as follows^59^:

13 $${{\rm{\Omega }}}_{{\rm{e}}x}[n]={{\rm{\Omega }}}_{{\rm{e}}x}[{n}_{0}]+{\mu }_{{\rm{e}}x}\int d{\bf{r}}\,{\rm{\Delta }}n({\bf{r}})-\frac{1}{2\beta }\int d{\bf{r}}\int d{\bf{r}}{\boldsymbol{^{\prime} }}\,{\rm{\Delta }}n({\bf{r}}){\rm{\Delta }}n({\bf{r}}{\boldsymbol{\text{'}}}){c}_{{\rm{e}}e}(|{\bf{r}}-{\bf{r}}{\boldsymbol{^{\prime} }}|)+O({n}^{2}),$$

where ${\rm{\Delta }}n({\bf{r}})=n({\bf{r}})-{n}_{0}$, and *μ*_ex_ is the excess chemical potential. Once the pair potential $v(q)$ has been specified, the self-consistent contributions of the excess free-energy functional can be calculated using the direct-correlation function via path integral quantum Monte Carlo (PIMC) simulations^60,61^, integral equations^62^ or analytical fits^63^. Let us now evaluate the free-energy functional of the non-interacting electron gas] *T*[*n*].

Many approximations for *T*[*n*]^64–66^, have been described in the literature- Thomas-Fermi (TF), Kirzhnits gradient correction (TFK)^65^, von Weizsäcker funtional (vW)^64^, Perrot functional^66^ to name a few. Most of these models are based on an extension of the TF functional

14 $${T}_{{\rm{T}}F}[n]=\frac{\sqrt{2}}{{\pi }^{2}{\beta }^{\mathrm{5/2}}}\int d{\bf{r}}\,[\alpha {I}_{\mathrm{1/2}}(\alpha )-\frac{2}{3}{I}_{\mathrm{3/2}}(\alpha )],$$

where

15 $${I}_{p}={\int }_{0}^{\infty }\frac{{x}^{p}}{1+{e}^{x-\alpha }}dx$$

is the Fermi-Dirac integral of order *p*, $\alpha (r)$ is the chemical potential normalized with the *k*_*B*_ *T* and the electron density is given by

16 $$n({\bf{r}})=\frac{\sqrt{2}}{{\pi }^{2}{\beta }^{\mathrm{3/2}}}{I}_{\mathrm{1/2}}[\alpha ({\bf{r}}\mathrm{)].}$$

We consider here the functional form for an electron gas based on the TF functional with the finite-temperature Kirzhnits gradient correction (TFK)^65^:

17 $${T}_{{\rm{T}}FK}[n]={T}_{{\rm{T}}F}[n]+\gamma \frac{3\sqrt{2}{\pi }^{2}}{8}{\beta }^{\mathrm{3/2}}\int d{\bf{r}}\frac{I{\text{'}}_{-\mathrm{1/2}}(\alpha )}{{I}_{-\mathrm{1/2}}^{2}(\alpha )}|\nabla n{|}^{2}+\cdots .$$

Here, we introduced a coefficient *γ* that allows to capture a variety of results. First, the systematic gradient expansion of Kirzhnits yields the prefactor *γ* = 1/9. Second, the von Weizsäcker result follows by a partial integration and *γ* = 1. The assumption in this gradient-correction expansion is that the error made by neglecting the third- and higher-order terms is very small. For high-density plasmas, interface-mixing problems or shock structures in which temperature and density gradients can be large, this expansion ceases to be valid. In such circumstances, it may be important to include higher-order terms for the thermal terms through higher gradient corrections *q* in the TFK functional. The non-interacting free-energy functional *T*[*n*] can also be expressed in terms of the Lindhard function, which is exactly known, instead of using OFDFT^66,67^. Let us now show the connection between DFT and the Bohm description. The functional derivative of the kinetic energy functional (17) is given by^20^

18 $$\frac{\delta {T}_{{\rm{T}}FK}}{\delta n}=\frac{\alpha (r)}{\beta }+\gamma \frac{3\sqrt{2}{\pi }^{2}}{8}{\beta }^{\mathrm{3/2}}\,[\xi ^{\prime} (\alpha )|\nabla n{|}^{2}+2\xi (\alpha ){\nabla }^{2}n],$$

where $\xi (\alpha )={I^{\prime} }_{-\mathrm{1/2}}(\alpha )/{I}_{-\mathrm{1/2}}^{2}(\alpha )$, its derivative with respect to the density is denoted ${\xi }^{\text{'}}(\alpha )$. The first term of (18) is the Fermi pressure while the second term corresponds to the generalized Bohm potential in the finite-temperature regime, revealing the underlying connection between DFT and the Bohm description. The connection between DFT and the Bohmian picture has recently been discussed in a paper by Stanton and Murillo^20^ where a Kirzhnits^65^ correction was used to get the “Bohm” term quite generally.

After linearizing and taking the Fourier transform of (13) and (17), the contributions of the non-interacting and interacting electron gases to the susceptibility become

19 $$\frac{\delta \tilde{V}({\bf{k}})}{\delta n({\bf{k}})}{|}_{0}=4\pi {\lambda }_{{\rm{T}}F}^{2}+\pi \nu {k}^{2}{\lambda }_{{\rm{T}}F}^{4}-{\beta }^{-1}{C}_{{\rm{e}}e}(k),$$

where the TF length and the parameter $\nu$ are given by

20 $${\lambda }_{{\rm{T}}F}^{2}=\frac{\pi \sqrt{2\beta }}{4{I}_{-\mathrm{1/2}}({\alpha }_{0})}\quad {\rm{and}}\quad \nu =\frac{\sqrt{8\beta }}{3\pi }{I}_{-\mathrm{3/2}}({\alpha }_{0}),$$

respectively. Substituting (19) into (12), we find

21 $${\chi }_{{\rm{e}}e}(q,\omega )=\frac{1}{4\pi {a}^{2}}\frac{{q}^{2}}{-{\omega }^{2}+{q}^{2}{\lambda }_{{\rm{T}}F}^{-2}+\frac{\nu }{4}{q}^{4}{\lambda }_{{\rm{T}}F}^{-4}-\frac{{q}^{2}}{3\Gamma }{n}_{0}{C}_{{\rm{e}}e}(q)-i{q}^{2}{\eta }_{l}\omega }\mathrm{.}$$

Here the frequency *ω* is in units of the electron plasma frequency, $q=|k|a$ is the wave number, $a={\mathrm{(3/4}\pi n)}^{\mathrm{1/3}}$ is the Wigner-Seitz radius, ${\mathop{\lambda }\limits^{ \mbox{-} }}_{{\rm{T}}F}={\lambda }_{{\rm{T}}F}/a$ is the Thomas-Fermi length in the units of the Wigner-Seitz radius, the viscosity is in units of $n{\omega }_{p}{a}^{2}$, and the coupling parameter, defined as the ratio of the potential energy to the average kinetic energy, is given by ${\rm{\Gamma }}=\beta /a$.

Substituting (21) into (5) yields the free-electron DSF

22 $$\frac{{\omega }_{p}{S}_{{\rm{e}}e}(q,\omega )}{n}=\frac{1}{\pi \sqrt{3{r}_{s}}}\frac{1}{1-\exp (-{\rm{\Gamma }}\sqrt{\mathrm{3/}{r}_{s}}\omega )}\frac{{q}^{4}{\eta }_{l}\omega }{{({\omega }^{2}+\frac{{q}^{2}}{3\Gamma }{n}_{0}{C}_{{\rm{e}}e}(q)-{q}^{2}{\lambda }_{{\rm{T}}F}^{-2}-\frac{\nu }{4}{q}^{4}{\lambda }_{{\rm{T}}F}^{-4})}^{2}+{({q}^{2}{\eta }_{l}\omega )}^{2}}\mathrm{.}$$

Equation (22) is the main result of this work. The second factor is the usual Bose function. The denominator of the third factor includes quantum degenerate plasma effects through the direct correlation function ${C}_{{\rm{e}}e}(q)$, thermal effects with high-order gradient terms, and viscous damping through ${\eta }_{l}$. It is worth noting that when the degeneracy parameter $\theta \sim {r}_{s}/{\rm{\Gamma }}$ is very large, electrons can be considered to be in a non-degenerate, classical state. If we then replace the exponential in the Bose function by its Taylor expansion, we recover the dynamic structure of non-degenerate electrons given by the Navier-Stokes model^68^.

The form of the DSF obtained from this theory, without the dissipative effects, is connected to the approaches based on the local field corrections^49^. The direct correlation function which is the main ingredient of this approach is related to the local field correction (LFC) $G(q)$ as:

23 $$G(k)=-1-\frac{1}{\beta v(k)}{C}_{{\rm{e}}e}(k\mathrm{).}$$

In the random phase approximation (RPA), the direct correlation function is given by ${G}_{{\rm{e}}{\rm{e}}}(k)=-\beta v(k)$ consequently the LFC vanishes, $G(k)=0$. Thus, the direct correlation function describes the strongly Coulomb correlation and exchange effects beyond the RPA. Several approximations for the LFC^45,46,49,69^, has been proposed starting from the formulation of local field corrections due to Coulomb correlations and exchange effects by Hubbard^45^. Utsumi and Ichimaru^49^ formula has been widely used to investigated the static properties of systems at metallic densities. Holas, Aravind and Singwi^70^ have suggested an expression for the dynamical LFC in strongly coupled electron gas. Although, we can use existing analytical fits for the LFC to obtain the direct correlation function, we choose here to computed this quantity directly using Ornstein-Zernike equations with the hypernetted-chain approximation closure^71^. Furthermore, matter under extreme conditions of temperature and pressure undergoes large spatial gradients (i.e., shocks structure, interface problems, etc.). The heterogeneity can greatly altered the Thompson spectra with respect to the uniform case as recently discussed by Kozlowski and coworkers^72^. In our approach, the constitutive equations will relax to the correct DFT thermodynamic ground state, which other methods cannot guarantee. This means we have the full *non-local* correlations absent from most other approaches opening up the possibility of studying the cases for which the usual homogeneous and isotropic forms like $\varepsilon ({\bf{k}},\omega )$ are not applicable. This can be done through the introduction of the inhomogeneous direct correlation function ${c}_{{\rm{e}}e}({\bf{r}},{\bf{r}}{\boldsymbol{\text{'}}})$. In the next section we will focus on the characteristic features of our main result (22).

## Discussion

Computing the DSF (22) requires knowledge of the viscosity and the direct correlation function. In the literature, the DSF is often expressed in terms of the local field correlation, and the latter quantity is evaluated using analytical fits^52,73,74^. The direct correlation function ${C}_{{\rm{e}}e}(q)$ can also be obtained directly through numerical simulations; that is the avenue pursued in this work, using hypernetted-chain calculations^62,75^, with a quantum statistical potential (QSP)^76–78^. We use QSP approach here to merely have easy access to results for which we can illustrate the DDFT method, which is the main point of the paper; other methods can be also used to get the structure information for the DDFT model. In fact, we see a strength of QSPs in this regard: the key quantity is the electron-electron ${c}_{{\rm{e}}e}(r)$, which is not accessible from DFT approaches. Electron-electron correlation functions are available from PIMC, however, and that provides validation for our input quantities. Jones and Murillo^79^ have shown the theoretical underpinnings of QSPs and reviewed their extension to fully degenerate quantum systems. Dutta and Dufty^60^ have compared compared QSP-based RDFs from the modified Kelbg QSP and PIMC and show that over an extremely wide range of physical conditions the QSP predictions are nearly perfect; it is only at very low densities that we can see a modest deviation. Here, we choose the QSP from the pioneering work of Hansen and McDonald^76^ since they yield results similar to the more complicated Kelbg potentials. Comparisons between Coulomb, HM and Kelbg potentials are shown in the online Supplementary Material. Next, we turn to the electronic viscosity is needed, which is determined by both electron-electron and electron-ion collisions. The electron viscosity is obtained by interpolating the zero-temperature viscosity proposed by Conti and Vignale^80^ and the finite-temperature viscosity for classical plasmas by Stanton and Murillo^33^. When building this fit, we considered contributions only from electron-electron interactions. However, the electron viscosity should also take into account electron-ion^81–83^, contributions, which can be more significant than the electron-electron viscosity in the regimes of interest. Please see the online Supplementary Material for a detailed description of the calculation of these two quantities.

**Figure 1:**
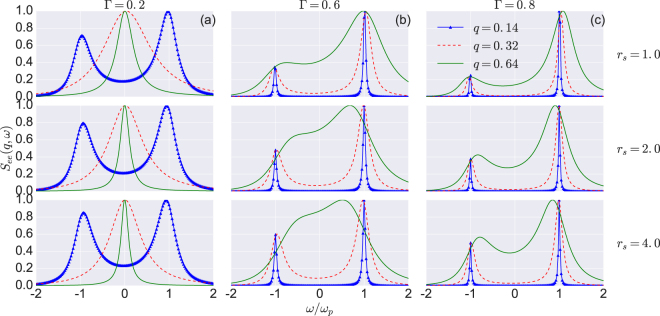
Effects of the coupling parameter on the spectra. We show the variation in the DSF for different values of the coupling parameter Γ and the normalized wavelengths $q=|{\bf{k}}|a$. The coupling parameter Γ ranges from 0.2 to 0.8, and *r*_*s*_ ranges from 1.0 to 4.0. The dynamic structure factor ${S}_{{\rm{e}}e}(q,\omega )$ is normalized by its maximum value. The two plasmon peaks are symmetric and the ratio of their amplitudes gives a measure of the electrons temperature.

Figure 1 shows the spectra of the DSF for different values of the wave number *q*, the coupling strength Γ and the density parameter *r*_*s*_. The free electron DSF is normalized by its maximum value. In Fig. 1a, the positions of the plasmon peaks (Stokes and anti-Stokes) and its amplitude remain almost unchanged when the quantum parameter *r*_*s*_ increases from 1.0 to 4.0. The reason is, in this regime $(\theta \gg \mathrm{1)}$, the correlations and quantum degeneracy effects are negligible and consequently have no impact on the propagation of the plasmon. Furthermore, the width of the plasmon peak reduces when *q* and *r*_*s*_ increase owing to the fact that the viscosity which acts to broaden the width of the peak is very sensitive to the density parameter *r*_*s*_. Figure 1a–c show that the position and width of the plasma peak vary strongly with Γ. These figures also display some of the standard features of the plasmons peaks; they are symmetric with respect to the zero frequency, and the difference between their amplitudes gives a measure of the electron temperature through the detailed balance relation. It is worth noting that for large values of the coupling parameter and density, the plasmon peak is at a frequency smaller than the plasma frequency ${\omega }_{p}$.

For a given value of the wave number *q*, the peak of ${S}_{{\rm{e}}e}(q,\omega )$ corresponds to the dispersion relation of the plasmon ${\omega }_{q}=\omega (q)$. According to (22), the dispersion relation is approximately given by

24 $${\omega }_{q}^{2}\cong {\omega }_{p}^{2}(-\frac{{q}^{2}}{3\Gamma }{n}_{0}{C}_{{\rm{e}}e}(q)+{\mathop{\lambda }\limits^{ \mbox{-} }}_{{\rm{T}}F}^{2}{q}^{2}+\frac{\nu }{4}{\mathop{\lambda }\limits^{ \mbox{-} }}_{{\rm{T}}F}^{4}{q}^{4}-\frac{{\eta }_{l}^{2}{q}^{4}}{2})\mathrm{.}$$

**Figure 2:**
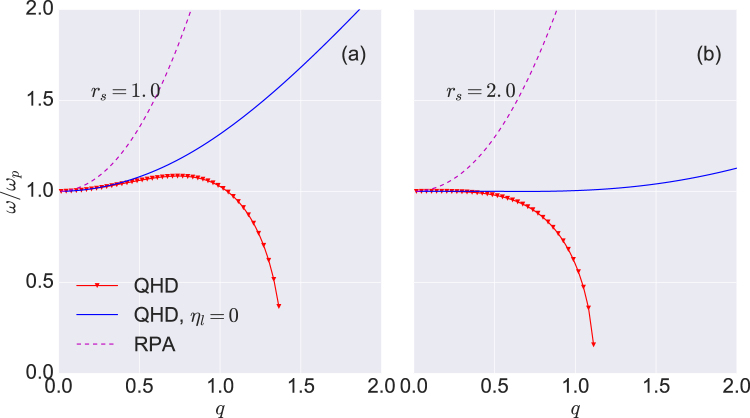
Plasmon dispersion relation. We show the frequency as a function of the wave number *q* for ${r}_{s}=1.86$ and (**a**) Γ = 1.0 and (**b**) Γ = 0.7. DDFT-QHD refers to (24), which accounts for strong correlations and viscosity. The label “DDFT-QHD: ${\eta }_{l}=0$” corresponds to (24) with the viscosity set to zero. The green curve shows the local field correction dispersion relation.

In the RPA limit, with a pure Coulomb potential, the direct correlation function is given by ${C}_{{\rm{e}}e}(q)=-\beta v(q)$. By substituting this result into (24) and setting the viscosity equal to zero, we recover the Bohm-Gross dispersion relation^84^ for an isothermal plasma. The dispersion relation of the plasmon is shown in Fig. 2 for ${r}_{s}=1.86$ and for Γ = 1.0 and Γ = 0.7. The red triangles lines show the DDFT-QHD result (24), and the data points indicated with blue line corresponds to (24), with the viscosity set to zero, *η*_*l*_ = 0. From our basic result (24) we can explore several limits that yield other models. For example, because the direct correlation function is related to the local-field correction through the relation ${c}_{ee}(q)=-\beta v(q\mathrm{)[1}-{G}_{ee}(q)]$, we can neglect the three higher-order terms in (24) to obtain the local-field correction (LFC) result. We show this limit in Fig. 2 as a green line. Next, we can retain the second and third terms to example quantum corrections to the LFC result, and that model is shown as blue triangles. The full result, including viscosity is shown as the red line; note that the viscous correction is large, suggesting that the power series in *q* of (24) is probably not converged at the largest values of *q* in the plot.

The dispersion relation (24) implies that the width of the peak of the DSF is broadened by the viscosity. Therefore, by measuring the width of the peak of a spectrum, information about the electron viscosity can be inferred. We can obtain a good estimate of the width in the following way. We know the location of the peak from the dispersion relation (24). Near that peak, we know what *ω* is, and this information can be put into ${q}^{2}{\eta }_{l}{\omega }_{peak}$ to obtain the width of the DSF. This scaling gives the width in terms of both *η*_*l*_ and ${C}_{{\rm{e}}e}(q)$, which means that *η*_*l*_ cannot be determined without knowledge of the direct correlation function. However, by fitting the entire spectrum of the DSF, all quantities can be obtained: the density, the temperature, and the viscosity. The DSF spectra suggest that measurements at a few *q* values is best.

## Concluding Remarks

A general framework for electron dynamics is provided with DDFT-QHD. Our model is the specific approximation of TDDFT in which the generalized-force functional is replaced by the equilibrium functional. We established the connection between DDFT-QHD and DSF through the fluctuation-dissipation theorem, allowing for improved QHD models to be compared with experimental data. The predicted DSF spectrum exhibits strong correlations and collisions that are built self-consistently into the model; this result differs from those obtained with more common Lindhard approaches^42–44^, in which collisions enter through a dynamic collision frequency^43,44^, or though local field corrections^85^. Our result suggests that the electronic viscosity can be determined experimentally by measuring the electron DSF.

Our approach is a full hydrodynamics model that can be used to simulate non-equilibrium, heterogeneous dense plasmas^72^. For example, we could investigate shock physics, fluid instabilities, and large-scale experiments. Most other methods are based on stationary, homogeneous/isotropic approximations; this is explicit in functions such as $\chi (k,\omega )$. Thus, while our DDFT formulation of QHD is significantly beyond these simple linear response functions, we show here that we are able to make contact with the XRTS community and connect the scattering spectrum to transport coefficients in a direct way with a hydrodynamic approach.

Finally, our model still lacks an equation for the energy fluctuations. Past experience suggests^54–57,86^, that energy fluctuations might cause a zero frequency mode. This latter was experimentally measured in a liquid lithium by Sinn and coworkers^87^ confirming molecular dynamics simulations results performed by Canales, GonzÃ¡lez, and Padró^86^. Our DDFT-QHD approach would miss any mode originating from thermal fluctuations^54,58,88^, because it is based on an isothermal assumption. We think this approach can incorporate an energy equation, but this is work in progress. In future work, it would be useful to include other transport quantities, such as the viscoelastic relaxation time and the thermal conductivity. Extension of this model to the full XRTS form factor with an electron-ion generalization of the DDFT-QHD equations^89,90^, is left for the future.

## Electronic supplementary material

Supplementary Information

## References

[1] Bostedt C (2016). Linac coherent light source: The first five years. Rev. Mod. Phys..

[2] Lindl J, Landen O, Edwards J, Moses E (2014). Review of the National Ignition Campaign 2009–2012. Phys. Plasmas.

[3] Saunders AM (2016). X-ray Thomson scattering measurements from hohlraum-driven spheres on the OMEGA laser. Rev. Sci. Instrum..

[4] Crowley BJB (2014). Continuum lowering - A new perspective. High Energy Density Physics.

[5] McKelvey, A. *et al*. Thermal conductivity measurements of proton-heated warm dense matter. In *APS Shock Compression of Condensed Matter Meeting Abstracts* (2015).

[6] Gregori G (2006). Measurement of carbon ionization balance in high-temperature plasma mixtures by temporally resolved X-ray scattering. J. Quant. Spectrosc. Radiat. Transf..

[7] Meezan NB (2017). Indirect drive ignition at the National Ignition Facility. Plasma Physics and Controlled Fusion.

[8] Thomas (2012). H. Explosions of Xenon Clusters in Ultraintense Femtosecond X-Ray Pulses from the LCLS Free Electron Laser. Phys. Rev. Lett..

[9] Hegelich BM (2006). Laser acceleration of quasi-monoenergetic MeV ion beams. Nature.

[10] Fletcher A, Close S, Mathias D (2015). Simulating plasma production from hypervelocity impacts. Physics of Plasmas.

[11] Bigot J-Y, Halté V, Merle J-C, Daunois A (2000). Electron dynamics in metallic nanoparticles. Chemical Physics.

[12] Wang Y, Eliasson B (2014). One-dimensional rarefactive solitons in electron-hole semiconductor plasmas. Phys. Rev. B.

[13] Davis P (2016). X-ray scattering measurements of dissociation-induced metallization of dynamically compressed deuterium. Nat. Commun..

[14] Bloch FB (1933). von Atomen mit mehreren Elektronen. Zeitschrift fur Physik.

[15] Hohenberg P, Kohn W (1964). Inhomogeneous electron gas. Phys. Rev..

[16] Mermin ND (1965). Thermal Properties of the Inhomogeneous Electron Gas. Physical Review.

[17] Ying SC (1974). Hydrodynamic response of inhomogeneous metallic systems. Nuovo Cimento B Serie.

[18] Gasser I, Jüngel A (1997). The quantum hydrodynamic model for semiconductors in thermal equilibrium. Zeitschrift Angewandte Mathematik und Physik.

[19] Michta D, Graziani F, Bonitz M (2015). Quantum Hydrodynamics for Plasmas - a Thomas-Fermi Theory Perspective. Contrib. Plasma Phys..

[20] Stanton LG, Murillo MS (2015). Unified description of linear screening in dense plasmas. Phys. Rev. E.

[21] Gardner CL (1994). Quantum hydrodynamic model for semiconductor devices. SIAM Journal of Applied Mathematics.

[22] Manfredi G, Haas F (2001). Self-consistent fluid model for a quantum electron gas. Phys. Rev. B.

[23] Levermore CD (1996). Moment closure hierarchies for kinetic theories. Journal of Statistical Physics.

[24] Degond P, Ringhofer C (2003). Quantum moment hydrodynamics and the entropy principle. Journal of Statistical Physics.

[25] Gardner CL (1994). Quantum hydrodynamic model for semiconductor devices. SIAM J. Appl. Math..

[26] Marini Bettolo Marconi U, Tarazona P (1999). Dynamic density functional theory of fluids. J. Chem. Phys.

[27] Lutsko JF (2008). Density functional theory of inhomogeneous liquids. III. Liquid-vapor nucleation. J. Chem. Phys..

[28] Diaw A, Murillo MS (2015). Generalized hydrodynamics model for strongly coupled plasmas. Phys. Rev. E.

[29] Runge E, Gross EKU (1984). Density-functional theory for time-dependent systems. Phys. Rev. Lett..

[30] Goddard BD, Nold A, Savva N, Pavliotis GA, Kalliadasis S (2012). General dynamical density functional theory for classical fluids. Phys. Rev. Lett..

[31] Marconi UMB, Tarazona P (1999). Dynamic density functional theory of fluids. J. Chem. Phys..

[32] Rex M, Löwen H (2008). Influence of hydrodynamic interactions on lane formation in oppositely charged driven colloids. Eur. Phys. J. E.

[33] Stanton LG, Murillo MS (2016). Ionic transport in high-energy-density matter. Phys. Rev. E.

[34] Perdew JP, Burke K, Ernzerhof M (1996). Generalized gradient approximation made simple. Phys. Rev. Lett..

[35] Malone FD (2016). Accurate exchange-correlation energies for the warm dense electron gas. Phys. Rev. Lett..

[36] Karasiev VV, Sjostrom T, Dufty J, Trickey SB (2014). Accurate homogeneous electron gas exchange-correlation free energy for local spin-density calculations. Phys. Rev. Lett..

[37] Huang C, Carter EA (2010). Nonlocal orbital-free kinetic energy density functional for semiconductors. Phys. Rev. B.

[38] Frenkel, J. *Kinetic Theory of Liquids* (Clarendon, Oxford, 1946).

[39] Glenzer SH, Redmer R (2009). X-ray thomson scattering in high energy density plasmas. Rev. Mod. Phys..

[40] Chihara J (2000). Interaction of photons with plasmas and liquid metals - photoabsorption and scattering. J. Phys. Condens. Matter.

[41] Sahoo S, Gribakin GF, Shabbir Naz G, Kohanoff J, Riley D (2008). Compton scatter profiles for warm dense matter. Phys. Rev. E.

[42] Mermin ND (1970). Lindhard dielectric function in the relaxation-time approximation. Phys. Rev. B.

[43] Thiele R (2010). Thomson scattering on inhomogeneous targets. Phys. Rev. E.

[44] Arkhipov YV, Davletov AE (1998). Screened pseudopotential and static structure factors of semiclassical two-component plasmas. Physics Letters A.

[45] Hubbard J (1957). The Description of Collective Motions in Terms of Many-Body Perturbation Theory. Proceedings of the Royal Society of London Series A.

[46] Singwi KS, Tosi MP, Land RH, Sjölander A (1968). Electron correlations at metallic densities. Phys. Rev..

[47] Vashishta P, Singwi KS (1972). Electron Correlations at Metallic Densities. V. Phys. Rev. B.

[48] Vaishya JS, Gupta AK (1973). Dielectric Response of the Electron Liquid in Generalized Random-Phase Approximation: A Critical Analysis. Phys. Rev. B.

[49] Utsumi K, Ichimaru S (1980). Dielectric formulation of strongly coupled electron liquids at metallic densities. II. Exchange effects and static properties. Phys. Rev. B.

[50] Geldart DJW, Vosko SH (1966). The screening function of an interacting electron gas. Canadian Journal of Physics.

[51] Dharma-wardana MWC, Perrot F (2000). Simple classical mapping of the spin-polarized quantum electron gas: Distribution functions and local-field corrections. Phys. Rev. Lett..

[52] Gregori G, Ravasio A, Höll A, Glenzer SH, Rose SJ (2007). Derivation of the static structure factor in strongly coupled non-equilibrium plasmas for X-ray scattering studies. High Energy Density Physics.

[53] Gregori G, Gericke DO (2009). Low frequency structural dynamics of warm dense mattera). Physics of Plasmas.

[54] Boon, J. P. & Yip, S. *Molecular hydrodynamics* (Dover Publications, New York, 1991).

[55] Pines, D. & Nozières, P. *The Theory of Quantum Liquids* (W. A. Benjamin, New York, 1989).

[56] Kugler AA (1973). Collective modes, damping, and the scattering function in classical liquids. Journal of Statistical Physics.

[57] Hansen JP, McDonald IR, Pollock EL (1975). Statistical mechanics of dense ionized matter. iii. dynamical properties of the classical one-component plasma. Phys. Rev. A.

[58] Schmidt R, Crowley BJB, Mithen J, Gregori G (2012). Quantum hydrodynamics of strongly coupled electron fluids. Phys. Rev. E.

[59] Hansen, J. & McDonald, I. *Kinetic Theory of Liquids* (Academic, London, 1986).

[60] Dutta S, Dufty J (2013). Uniform electron gas at warm, dense matter conditions. EPL (Europhysics Letters).

[61] Brown EW, Clark BK, DuBois JL, Ceperley DM (2013). Path-Integral Monte Carlo Simulation of the Warm Dense Homogeneous Electron Gas. Phys. Rev. Lett..

[62] Xu H, Hansen J-P (1998). Density-functional theory of pair correlations in metallic hydrogen. Phys. Rev. E.

[63] Groth, S., Dornheim, T. & Bonitz, M. Free Energy of the Uniform Electron Gas: Testing Analytical Models against First Principle Results. *ArXiv e-prints* (2016).

[64] Weizsäcker CFV (1935). Zur Theorie der Kernmassen. Zeitschrift fur Physik.

[65] Kirzhnits D (1957). Quantum Corrections to the Thomas-Fermi Equation. *ZSoviet Phys*. JETP.

[66] Perrot F (1994). Hydrogen-hydrogen interaction in an electron gas. J. Phys.: Cond. Mat..

[67] Wang L-W, Teter MP (1992). Kinetic-energy functional of the electron density. Phys. Rev. B.

[68] Murillo MS (2010). X-ray thomson scattering in warm dense matter at low frequencies. Phys. Rev. E.

[69] Farid B, Heine V, Engel GE, Robertson IJ (1993). Extremal properties of the harris-foulkes functional and an improved screening calculation for the electron gas. Phys. Rev. B.

[70] Holas A, Aravind PK, Singwi KS (1979). Dynamic correlations in an electron gas. I. First-order perturbation theory. Phys. Rev. B.

[71] Wünsch K, Hilse P, Schlanges M, Gericke DO (2008). Structure of strongly coupled multicomponent plasmas. Phys. Rev. E.

[72] Kozlowski PM, Crowley BJB, Gericke DO, Regan SP, Gregori G (2016). Theory of Thomson scattering in inhomogeneous media. Scientific Reports.

[73] Ichimaru S (1993). Nuclear fusion in dense plasmas. Rev. Mod. Phys..

[74] Nagao K, Bonev SA, Ashcroft NW (2001). Cusp-condition constraints and the thermodynamic properties of dense hot hydrogen. Phys. Rev. B.

[75] Chihara J (1991). Unified description of metallic and neutral liquids and plasmas. J. Phys. Condens. Matter.

[76] Hansen JP, McDonald IR (1981). Microscopic simulation of a strongly coupled hydrogen plasma. Phys. Rev. A.

[77] Schwarz V (2010). Static ion structure factor for dense plasmas: Semi-classical and ab initio calculations. High Energ. Dens. Phys..

[78] Lado F (1967). Effective Potential Description of the Quantum Ideal Gases. J. Chem. Phys..

[79] Jones CS, Murillo MS (2007). Analysis of semi-classical potentials for molecular dynamics and Monte Carlo simulations of warm dense matter. High Energy Density Physics.

[80] Conti S, Vignale G (1999). Elasticity of an electron liquid. Phys. Rev. B.

[81] Murillo MS (2008). Viscosity estimates of liquid metals and warm dense matter using the Yukawa reference system. High Energ. Dens. Phys..

[82] Clérouin J (2002). The viscosity of dense hydrogen: from liquid to plasma behaviour. J. Phys. Condens. Matter.

[83] Faussurier G, Libby SB, Silvestrelli PL (2014). The viscosity to entropy ratio: From string theory motivated bounds to warm dense matter transport. High Energ. Dens. Phys..

[84] Gouedard C, Deutsch C (1978). Dense electron-gas response at any degeneracy. Journal of Mathematical Physics.

[85] Ichimaru S, Tanaka S (1986). Generalized viscoelastic theory of the glass transition for strongly coupled, classical, one-component plasmas. Phys. Rev. Lett..

[86] Canales M, González LE, Padró JÀ (1994). Computer simulation study of liquid lithium at 470 and 843 K. Phys. Rev. E.

[87] Sinn H (1997). Coherent dynamic structure factor of liquid lithium by inelastic x-ray scattering. Phys. Rev. Lett..

[88] Mountain RD (1966). Spectral distribution of scattered light in a simple fluid. Rev. Mod. Phys..

[89] Fu Z-G (2016). Dynamic properties of the energy loss of multi-mev charged particles traveling in two-component warm dense plasmas. Phys. Rev. E.

[90] Barriga-Carrasco MD (2008). Target electron collision effects on energy loss straggling of protons in an electron gas at any degeneracy. Physics of Plasmas.

