# Supplementary material for: A viscous quantum hydrodynamics model based on dynamic density functional theory
Source: Sci Rep. 2017 Nov 10;7:15352. doi: 10.1038/s41598-017-14414-9 (PMC5681597; doi:10.1038/s41598-017-14414-9)
Supplement: Supplementary file 1 — Supplementary Information [file 41598_2017_14414_MOESM1_ESM.pdf]

Supplementary Material : A viscous quantum hydrodynamics model based on Dynamic Density Functional Theory

Abdourahmane Diaw, Michael S. Murillo

Department of Computational Mathematics, Science and Engineering, Michigan State University, East Lansing 48823, USA

Appendix

Our viscous QHD formulation requires knowledge of both the direct correlation function $C_{ee}(q)$ and the electronic longitudinal viscosity η_l. In this appendix we describe the methodology used to obtain these quantities.

The radial distribution function for a *classical* system with pair interactions $u(r)$ can be found by solving the equations

$$g(r) = \exp\left[-\beta u(r) + h(r) - c(r) + B(r)\right], \tag{1}$$

$$h(r) = c(r) + n_0 \int c(|\mathbf{r}-\mathbf{r}'|)h(r')d\mathbf{r}', \tag{2}$$

which are the closure and the Ornstein-Zernicke equation (OZE), respectively. While these are exact, the bridge function $B(r)$ is not known for a limited number of systems[1]. However, the electron-electron coupling is modest in most physical regimes of interest[2], and we can neglect $B(r)$, which is the so-called hypernetted chain (HNC) approximation, a closed set of equations for $c(r)$ and $h(r) = g(r)-1$. Here, β is the inverse temperature in energy units, n_0 is the mean density, and $c(r)$ is the direct correlation function. Fast algorithms for solving the HNC equations over a wide range of parameters have been discussed elsewhere[3,4]. When constructing such algorithms, it is essential to remove the long-range character of the functions, choose an iteration cycle beginning with a reasonable initial guess, and solve the OZE in Fourier space. From the self-consistent solution, we can find the direct correlation function through

$$C(q) = \frac{H(q)}{1+n_0 H(q)}. \tag{3}$$

We can examine the role of strong coupling by comparing the numerical solution of the HNC equations with the analytical solution of their mean-field approximations, obtained with $c_{mf}(r) = -\beta u(r)$.

Of course, we require the correlation functions for the *quantum* system, and under conditions of modest to large degeneracy. The classical HNC scheme is readily modified to treat the quantum case if quantum statistical potentials (QSPs) are used in place of $u(r)$[5]. Such an approach guarantees that $g(r) > 0, \forall r$. Dutta and Dufty[6] have compared the modified Kelbg QSP with the gold standard of PIMC[1]; their results show that over an extremely wide range of physical conditions the QSP and PIMC results are in near perfect agreement, except at very low densities where there is a quite modest deviation. Here, we employ both diffraction and Pauli exclusion potentials, using the Hansen and McDonald[7] form

$$u(r) = \frac{1}{r}\left[1-\exp\left(\frac{-r}{\lambda_{ee}}\right)\right] + \beta^{-1}(\ln 2)\exp\left(\frac{-r^2}{\pi\lambda_{ee}^2(\ln 2)}\right), \tag{4}$$

where the DeBroglie thermal wavelength is defined as

$$\left(\frac{\lambda_{ee}}{a}\right)^2 = \frac{\Gamma}{\pi r_s} = \frac{2}{\pi}\left(\frac{4}{9\pi}\right)^{2/3}\frac{1}{\theta}. \tag{5}$$

Here a is the Wigner-Seitz radius, $\Gamma = \beta/a$ is the coupling parameter, $r_s = a/a_B$ is the density parameter, a_B is the Bohr radius, and θ is the quantum degeneracy defined as β_F/β where β_F is the inverse of the Fermi temperature.

Results from the HNC-QSP model can be obtained analytically in the mean-field approximation. Numerical results are shown in Fig. 1, where we compare the HNC and mean-field approximations for a range of Γ and $r_s = 2$; as expected, the mean-field result differs from the HNC result when $\Gamma \gtrsim 1$. Moreover, the difference is quite small, further justifying the neglect of $B(r)$.

Figure 2 displays the spatial profile of HM, Kelgb, modified-Kelgb and pure Coulomb potentials. The comparison between these different QSPs show good agreement except at small r for some cases. For $r > 1$, we see some lack of sensitivity of the choice of the potential, suggesting that most QSPs will yield results close to PIMC results. However, we compare our model with PIMC[8] results in Fig. 3; except for very strong degeneracy $\theta = 0.5$, our model is in quite reasonable agreement with PIMC, confirming the results of Dutta and Dufty.

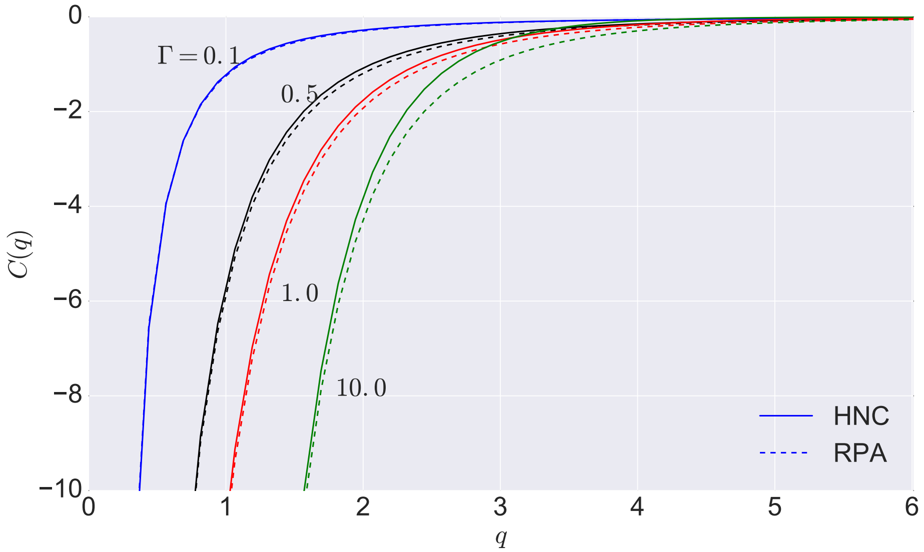

Figure 1. Predicted direct correlation function (DCF). Predictions of the DCF for $r_s = 2$ and $\Gamma = 0.1, 0.5, 1.0, 10$ from the QSP-HNC model and its mean-field approximation.

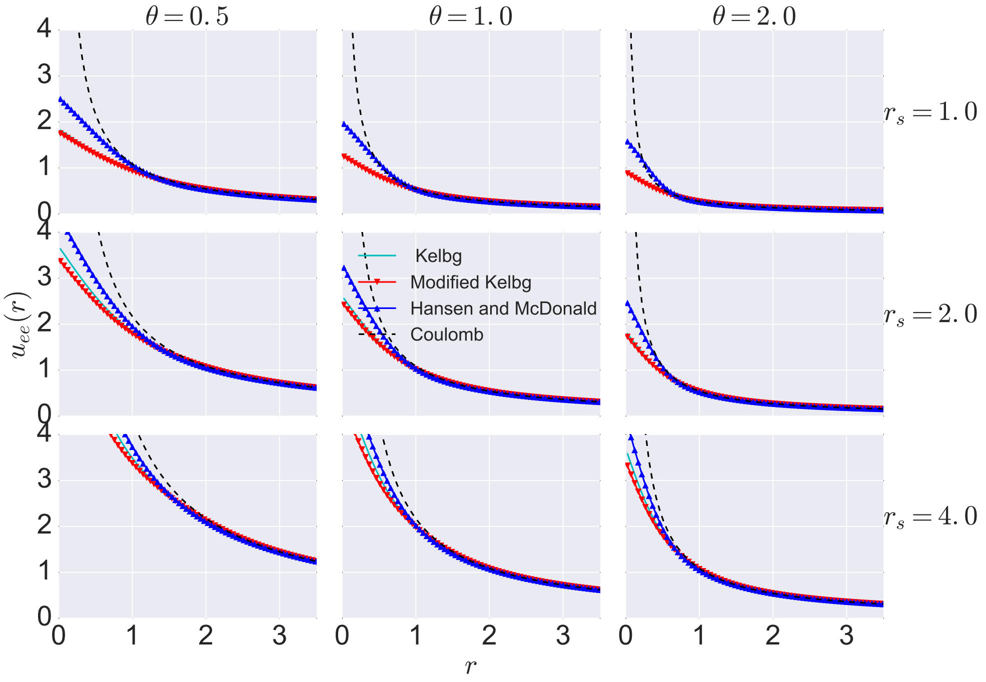

Figure 2. Comparison of quantum statistical potential for different densities and temperature. Electron-electron QSPs obtained with Kelbg[9], modified-Kelbg[6], Hansen and McDonald quantum[7] statistical potentials and the pure Coulomb potential for different values of θ and r_s. There are small r differences at high density.

Next, to determine the longitudinal viscosity $\eta_l = (4\eta/3 + \xi)$ which contains the shear η and bulk ξ viscosities of the electron fluid, we proceed as follows. In the zero-temperature limit, Conti and Vignale[10] suggested that the electron shear

[1] It is worth emphasizing that $g(r)$ here refers to the electron-electron $g_{ee}(r)$, a quantity not readily obtainable from DFT approaches.

viscosity in the units of $na^2\omega_p$ can be approximated by

$$\eta_{CV}^* = \frac{1}{\sqrt{3r_s}} \frac{1}{c_0 r_s^{-3/2} + c_1 r_s^{-1} + c_2 r_s^{-2/3} + c_3 r_s^{-1/3}}, \tag{6}$$

where $c_0 = 60, c_1 = 80, c_2 = -40$, and $c_3 = 62$. In the classical limit, Stanton and Murillo[11] proposed an accurate fit for the

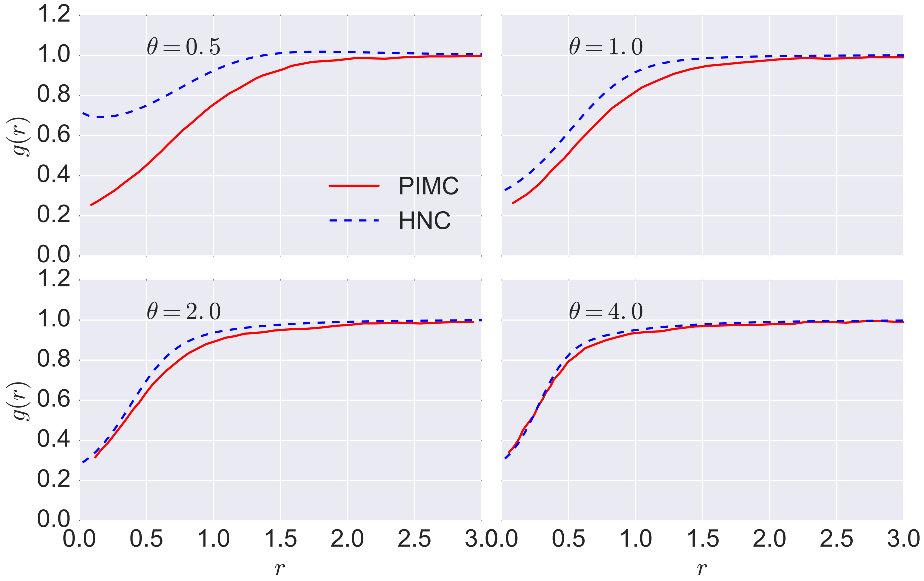

Figure 3. Comparison between HNC-QSP and PIMC for $g(r)$ at four values of the degeneracy parameter $\theta = T/E_F$. The QSPs used are the Hansen and McDonald[7] forms and the PIMC is from Brown[8].

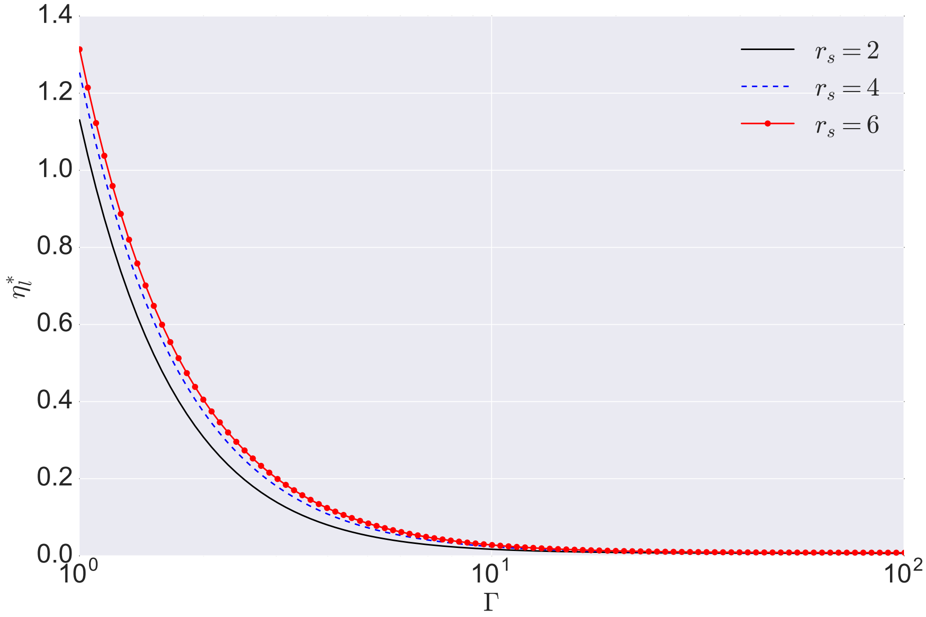

Figure 4. Electronic viscosity. We show the viscosity (8) as a function of the coupling parameter Γ for different values of r_s.

electron viscosity:

$$\eta_{SM}^* = \frac{5\sqrt{3\pi}}{18\Gamma^{5/2}\ln\Lambda_\eta}, \tag{7}$$

where the coupling parameter is defined as $\Gamma = \beta/a$, and Λ_η is the effective Coulomb logarithm for viscosity, for which a numerical solution is given in Ref.[11]. In our calculation, we use the Thomas-Fermi length as the screening length. We obtain the viscosity in both the zero- and finite- temperature limits by interpolating the two formulas (6) and (7) as

$$\eta_l^* = \frac{4}{3}\frac{\eta_{CV}^* + \theta\eta_{SM}^*}{1+\theta}, \tag{8}$$

where the degeneracy parameter is $\theta = T/T_F$, and T_F is the Fermi temperature. Note that the bulk viscosity ξ has been shown to vanish identically both in quantum[12] and classical fluids[13]. The result of the interpolation for the viscosity is shown in Fig. 4 as a function of the coupling parameter.

References

1. Daughton, W., Murillo, M. S. & Thode, L. Empirical bridge function for strongly coupled yukawa systems. *Phys. Rev. E* **61**, 2129–2132 (2000).
2. Stanton, L. G. & Murillo, M. S. Unified description of linear screening in dense plasmas. *Phys. Rev. E* **91**, 033104 (2015).
3. Springer, J. F., Pokrant, M. A. & Stevens, F. A., Jr. Integral equation solutions for the classical electron gas. *J. Chem. Phys.* **58**, 4863–4867 (1973).
4. Ng, K.-C. Hypernetted chain solutions for the classical one-component plasma up to Gamma equals 7000. *J. Chem. Phys.* **61**, 2680–2689 (1974).
5. Jones, C. S. & Murillo, M. S. Analysis of semi-classical potentials for molecular dynamics and Monte Carlo simulations of warm dense matter. *High Energy Density Physics* **3**, 379–394 (2007).
6. Dutta, S. & Dufty, J. Uniform electron gas at warm, dense matter conditions. *EPL (Europhysics Letters)* **102**, 67005 (2013).
7. Hansen, J. P., McDonald, I. R. & Pollock, E. L. Statistical mechanics of dense ionized matter. iii. dynamical properties of the classical one-component plasma. *Phys. Rev. A* **11**, 1025–1039 (1975).
8. Brown, E. W., Clark, B. K., DuBois, J. L. & Ceperley, D. M. Path-integral monte carlo simulation of the warm dense homogeneous electron gas. *Phys. Rev. Lett.* **110**, 146405 (2013).
9. Graziani, F. R. *et al.* Large-scale molecular dynamics simulations of dense plasmas: The Cimarron Project. *High Energy Density Physics* **8**, 105–131 (2012).
10. Conti, S. & Vignale, G. Elasticity of an electron liquid. *Phys. Rev. B* **60**, 7966–7980 (1999).
11. Stanton, L. G. & Murillo, M. S. Ionic transport in high-energy-density matter. *Phys. Rev. E* **93**, 043203 (2016).
12. Nifosi, R., Conti, S. & Tosi, M. P. Dynamic exchange-correlation potentials for the electron gas in dimensionality D=3 and D=2. *Phys. Rev. B* **58**, 12758–12769 (1998).
13. Vieillefosse, P. & Hansen, J. P. Statistical mechanics of dense ionized matter. v. hydrodynamic limit and transport coefficients of the classical one-component plasma. *Phys. Rev. A* **12**, 1106–1116 (1975).
